# Could Cells from Your Nose Fix Your Heart? Transplantation of Olfactory Stem Cells in a Rat Model of Cardiac Infarction

**DOI:** 10.1100/tsw.2010.40

**Published:** 2010-03-16

**Authors:** Cameron McDonald, Alan Mackay-Sim, Denis Crane, Wayne Murrell

**Affiliations:** ^1^ Eskitis Institute for Cell and Molecular Therapies, Griffith University, Nathan, QLD, Australia; ^2^ School of Biomolecular and Physical Sciences, Griffith University, Nathan, QLD, Australia; ^3^ National Centre for Adult Stem Cell Research, Griffith University, Nathan, QLD, Australia

**Keywords:** olfactory, stem cell, cardiac repair, infarction, rat model, cell transplant

## Abstract

This study examines the hypothesis that multipotent olfactory mucosal stem cells could provide a basis for the development of autologous cell transplant therapy for the treatment of heart attack. In humans, these cells are easily obtained by simple biopsy. Neural stem cells from the olfactory mucosa are multipotent, with the capacity to differentiate into developmental fates other than neurons and glia, with evidence of cardiomyocyte differentiation *in vitro* and after transplantation into the chick embryo. Olfactory stem cells were grown from rat olfactory mucosa. These cells are propagated as neurosphere cultures, similar to other neural stem cells. Olfactory neurospheres were grown *in vitro*, dissociated into single cell suspensions, and transplanted into the infarcted hearts of congeneic rats. Transplanted cells were genetically engineered to express green fluorescent protein (GFP) in order to allow them to be identified after transplantation. Functional assessment was attempted using echocardiography in three groups of rats: control, unoperated; infarct only; infarcted and transplanted. Transplantation of neurosphere-derived cells from adult rat olfactory mucosa appeared to restore heart rate with other trends towards improvement in other measures of ventricular function indicated. Importantly, donor-derived cells engrafted in the transplanted cardiac ventricle and expressed cardiac contractile proteins.

### INTRODUCTION

There is widespread enthusiasm for the prospect of some kind of cellular transplant therapy for repair of failing organs[1,2]. We examine here the prospect of autologous tissue repair for heart attack. We have used a rat model of cardiac infarction in order to undertake a pilot study to test the feasibility of using a patient's own olfactory stem cells as a cellular source. Using a patient's own neural stem cells derived by simple biopsy and expanded *in vitro* to supply cellular substrate for the assisted healing of heart muscle tissue after infarct would seem an attractive scenario.

### Cardiogenic Potential of Olfactory-Derived Stem Cells

The olfactory epithelium must undergo vigorous neurogenesis and continual replacement of sensory neurons throughout adult life. We work on the potential use of the putative olfactory stem cell in tissue transplant therapies and have demonstrated a broad multipotency of these cells[3]. As well, they have been demonstrated to be therapeutic in a rat model of Parkinson's disease[4], where human olfactory neural stem cells were transplanted into rat brains. Another pilot study demonstrated their ability to express chondrogenic phenotype *in vitro* and after transplant in a rat intervertebral disc model[5]. They are an attractive source of autologous stem cells because of the ease with which they can be obtained by simple biopsy of as little as 1 $mm^3$ of tissue from a patient's nose[3]. In an *in vitro* transwell induction experiment, cells expressed two cardiac muscle–specific protein markers[3]. In a chick embryo transplant model, olfactory-derived cells (both adult human and adult mouse) were integrated into beating heart muscle and subsequently confirmed to express cardiac-specific protein[3].

In 1994, transplantation of syngeneic fetal cardiomyoblasts in mouse myocardium led to stable, functional engraftment[6], demonstrating the potential for the repair of cardiac tissue using cell transplantation therapy and leading to a number of human clinical trials[7,8]. A variety of cell types have been transplanted in animal models of cardiac infarction: embryonic stem cells (ES)[9,10,11], predifferentiated ES cells[9], fetal skeletal myoblasts[10,12], bone marrow–derived stem cells[13,14,15,16] (variously described as hematopoietic, stromal, mesenchymal, and endothelial[17,18,19,20,21]), and cardiac-derived stem cells[7,9,22,23]. Functional improvement is demonstrated in many cases, but evidence of a cellular mechanism is often lacking because of the difficulty in the tracking of donor-derived cells[24,25]. In the case of nonautologous cell source (embryonic or fetal), the issue of transplant histocompatibility remains a significant hurdle[26]. In recent times, the isolation of multipotent stem cells has been reported from several adult tissues, including bone marrow[27,28,29], brain[30,31,32,33], skeletal muscle[34], adipose tissue[35], dermis[36], olfactory mucosa[3], and even myocardium[37,38,39]. These reports suggest that stem-like cells exist within many adult tissues and organs, and that these cells may be pluripotent, with a developmental potential perhaps comparable to that of embryonic-derived stem cells and, importantly, may provide a basis for the development of autologous cell transplant therapy. An indication of cardiac myogenicity was demonstrated initially in early embryo transplant experiments, where adult-derived stem cells (from both neural and bone marrow sources) were seen to have contributed to a functioning myocardium in developing chick or mouse embryos[29,32]. Acquisition of cardiac lineage is believed to involve initiation of Wnt signaling[40]. To date, virtually all adult-derived donor cells in heart repair experiments have been from skeletal muscle, bone marrow, blood, or described as being of mesenchymal phenotype. This is the first report of experimental therapy using adult neural stem cells.

## MATERIALS AND METHODS

### Experimental Plan

Olfactory stem cells were grown from rat olfactory mucosa. These cells are propagated as neurosphere cultures[3], similar to other neural stem cells[30]. Olfactory neurospheres were grown *in vitro*, dissociated into single cell suspensions, and transplanted into the infarcted heart of congeneic rats. Donor cells and recipient animals were of the highly inbred Dark Agouti (DA) rat strain in order to emulate autologous cell transplantation. Transplanted cells were genetically engineered to express green fluorescent protein (GFP) in order to allow them to be identified after transplantation. Functional assessment was made using echocardiography in three groups of rats: control, unoperated; infarct only; infarcted and transplanted. Histological assessment confirmed engraftment of transplanted cells and their expression of myocardial phenotype.

### Animals

All animals were of the inbred DA strain of rat obtained from the Animal Resources Centre (Perth, Western Australia) and housed in the Griffith University Animal Housing Facility (Queensland, Australia). Animal housing, handling, and experimental use were all in accordance with the guidelines of Griffith University Animal Ethics Committee and the National Health and Medical Research Council of Australia. The investigation conforms with the "Guide for the Care and Use of Laboratory Animals" published by the U.S. National Institutes of Health (NIH Publication No. 85-23, revised 1996).

### Cell Preparation and Neurosphere Culture

Rats were killed with an anesthetic overdose (pentobarbitone sodium, >0.1 mg/g bodyweight) and olfactory mucosa was prepared as previously described[3,41]. Mucosal biopsies were immediately placed on ice in DMEM/HAM F12 medium (Invitrogen) supplemented with 10% fetal calf serum (FCS), penicillin, and streptomycin, and incubated for 45 min at 37°C in a 2.4 units/ml dispase II solution (Boehringer). The lamina propria was carefully separated from the epithelium under a dissection microscope with a microspatula. The cells of the olfactory epithelium were mechanically dissociated by trituration. The lamina propria was cut into pieces of approximately 40 $\mu m^2$ using a McIlwain chopper (Brinkmann) and incubated in a 0.25% Collagenase IA solution (Sigma) made up in DMEM/HAM F12 supplemented with ITS (insulin [10 μg/ml], transferrin [5.5 μg/ml], sodium selenite [6.7 ng/ml], and sodium pyruvate [110 μg/ml]), and penicillin (100 U/ml) and streptomycin (100 μg/ml)]. The dissociated cells of the lamina propria and epithelium were combined and plated on uncoated plastic dishes, and grown as primary cultures in DMEM/FCS until confluence, usually 8–12 days. At confluence, the cells were washed twice with HBSS (Hank's Balanced Saline Solution, JRH Biosciences) to remove serum products, released from the culture dish with trypsin/EDTA (solution of 0.25% trypsin, 0.02% ethylenediaminetetraacetic acid in HBSS), and incubated at 37°C in 5% $CO_2$ for 10 min, after which DMEM/FCS was added to inhibit the trypsin). The detached cells were then collected, centrifuged, and resuspended in DMEM/ITS. Cell counting was undertaken in triplicate using a hemocytometer to ensure an accurate estimate of cell numbers. The cells were subsequently plated onto six-well plastic plates coated with poly-L-lysine (PLL, 0.85 $\mu g/cm^2$ for 3–4 h) and grown in DMEM/ITS containing basic fibroblast growth factor (25 ng/ml, Sigma) and epidermal growth factor (50 ng/ml, Sigma). Cells under these latter conditions grow as multipotent neurospheres, the same as those described in our paper[3].

For the analysis of survival and phenotype of transplanted cells, olfactory neurosphere-derived cells were labeled with GFP as described[3]. Briefly, transduction experiments used a pFB-hrGFP, Viraport™ (Stratagene) retroviral control supernatant that contains an MMLV replication-defective retrovirus. As a precaution, cells targeted for transduction were tested using a reverse transcriptase assay in case of endogenous replication-competent retrovirus. When confirmed to be clear of endogenous retrovirus, they were transduced according to the manufacturer's instructions. PCR was then used to confirm genomic integration of the viral insert. Neurospheres obtained from GFP-transduced cultures were tested capable of producing both neurons and glia prior to use in transplant experiments. Cells were selected (>90%) for strong GFP expression using fluorescence-activated cell sorting (FACs Aria, Becton Dickenson), and subsequently cultured as neurospheres and maintained in culture until required for transplantation. Neurospheres were dissociated in trypsin (0.25% trypsin, 0.02% EDTA) until an even cell suspension was obtained. The cells were resuspended in HBSS supplemented with 2% FCS and counted, after which the suspension volume was adjusted to provide 20,000 cells/μl.

### Cardiac Infarction and Cell Transplantation

Prior to surgery, animals (19 female rats; 10–12 weeks; 140–150 g) were given preoperative valium (7-chloro-1, 3-dihydro-1-methyl-5-phenyl-2H-1, 4-benzodiazepin-2-one, 0.75 mg i.p.). Thirty minutes later, the animals were placed into an anesthetic flow chamber with a constant flow of 4% isofluorane in medical oxygen until sedated. Topical xylocaine (2-(diethylamino)-*N*-(2,6-dimethylphenyl)acetamide) was applied to the operative skin sites and the skin topically disinfected (Betadine). After intubation, the animals were connected to a ventilator to deliver 65 breaths per minute, tidal volume 0.2 ml. Surgical anesthesia was subsequently adjusted with 2–3% isofluorane according to animal monitoring throughout the surgical procedure. Chloromycetin ointment was applied to the eyes to prevent drying or infection. Body temperature was regulated throughout the procedure via a heating pad and rectal thermometer.

The heart was exposed by incision between the fifth and sixth ribs on the left-hand side of the animal, allowing visualization of the beating heart (Fig. 1). The heart was exteriorized using forceps and the main coronary artery identified. Electrocauterization was used to cut and seal the lower anterior left coronary artery, effectively occluding blood flow to the lowest third of ventricular height. Attention was paid to making the position and extent of the occlusion as uniform as possible for all animals so treated (Fig. 2A). This region was monitored for blanching to confirm occlusion of the blood supply and the border region of the forming infarct was identified. Following the infarct, topical lidocaine (2-(diethylamino)-*N*-(2,6-dimethylphenyl)acetamide) was applied to the region of insult to decrease the probability of fibrillation.

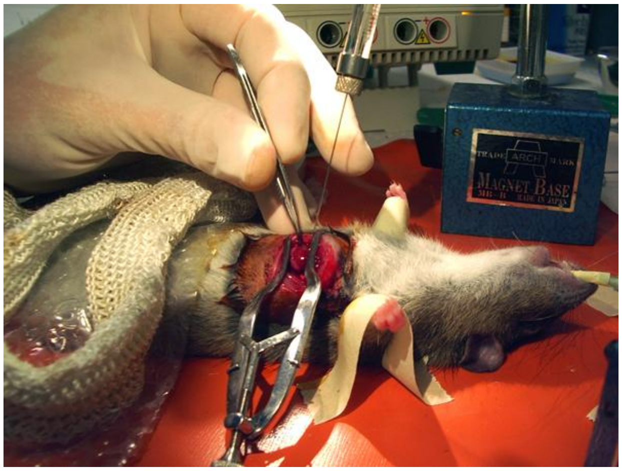

**FIGURE 1.** The heart was exteriorized using forceps and the main coronary artery identified. Electrocauterization was used to cut and seal the lower anterior left coronary artery, effectively occluding blood flow to the lowest third of ventricular height. Five microliters of the olfactory cell suspension was injected into each of five sites around the border region of the infarct using a Hamilton syringe.

Five microliters of the olfactory cell suspension was injected into five sites around the border region of the infarct using a Hamilton syringe (Fig. 2A). Care was taken to inject the cells into the ventricular wall and not right through into the ventricular chamber. A total of 25-μl volume containing approximately 500,000 cells was injected into each infarcted heart. The heart was then returned to the chest cavity and the ribs, overlaying muscles, and skin were closed with dissolvable sutures. As part of this procedure the chest was temporarily intubated through the wound to evacuate fluids after the surgery.

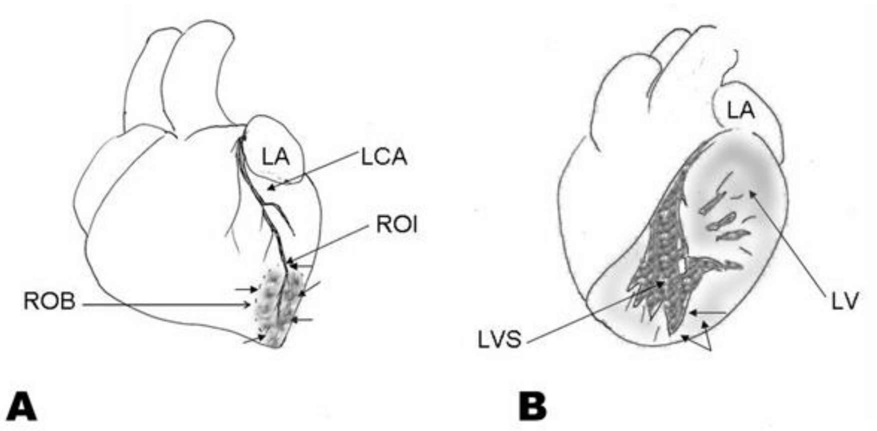

**FIGURE 2**. (A) Diagram showing the site of occlusion and of transplant. (B) Cutaway of left ventricle indicating regions sampled for histology. LA, left atrium; LCA, left coronary artery; ROI, region of infarct; ROB, region of blanching. Small arrows, sites of cell injection. (B) Cutaway of left ventricle. LA, left atrium; LV, left ventricle (muscle sagittal cutaway); LVS, left ventricular space. Small arrows, regions sectioned for immunochemistry.

### Postsurgical Cardiac Function Testing

Animals receiving infarcts were not assessed for cardiac performance immediately postinfarct as the severity of surgery required ideal postoperative conditions. Animals were allowed to recover for 4 weeks postsurgery. Echocardiography was performed by qualified pediatric echocardiographers using a pediatric scanner at the Prince Charles Hospital, Brisbane. During this procedure, animals were sedated (ketamine, 50 mg/kg, i.p.). Fifteen animals were assessed with functional echocardiography: control group, six animals; infarct only group, four animals; infarct then transplanted group, five animals. Due to the technical limitations, data points were obtained for only 18 of 20 parameters measured for all three groups, which were compared using multivariate analysis of variance to identify any group differences.

### Histological Assessment of Engraftment

One week following functional testing, 35 days postinfarct, the transplanted animals were euthanized by lethal injection (pentobarbitone sodium, >0.1 mg/g bodyweight). After death, the heart was dissected from the chest and sliced into transverse slices of 4–5 mm. These slices were then washed in PBS for 5 min, following which they were fixed by submersion in 4% paraformaldehyde in PBS under vacuum for 2 h. Slices were then washed again in PBS and equilibrated in 10% sucrose in PBS overnight. Slices were then frozen in OTC and sectioned transversely at 8 μm. Sections were then stored at –80°C until used.

Immunofluorescent techniques for antigen detection were not used due to the high degree of autofluorescence characteristic of fixed cardiac muscle that has led to some recent experimental misinterpretation. Transplanted cells were identified within sections using a goat anti-GFP antibody (Santa Cruz Biotechnology Inc. SC-5385) at a dilution of 1:100. The secondary antibody was biotinylated horse antigoat IgG (1:400, Vector) and detected using ABC kit (Vector Labs) with 3,3'-diaminobenzidine (DAB). Briefly, sections were blocked and permeablized in 10% serum of the animal used to raise the secondary antibody in 1% Triton X100, 0.3% $H_2O_2$ (to extinguish endogenous peroxidase). DAB detection took place in the presence of 0.3% $NiCl_2.6H_2O$ in order to achieve black coloration. Sections omitting secondary antibody ensured the absence of effects of endogenous biotin and sections omitting ABC reagents ensured the absence of effects of endogenous peroxidase.

For the subsequent detection of phenotypic markers, primary antibodies were applied in a range of concentrations; however, secondary decoration used a goat antimouse alkaline phosphatase–conjugated antibody (Bioscience) at 1:200 for enzymatic conversion of Sigma Fast Red. Primary antisera used were mouse antisarcomeric tropomyosin (Sigma T9283), mouse anticardiac troponin I (Chemicon MAB3150), mouse antisarcomeric α-actin (Sigma A2172), and mouse anti-GATA 4 (Santa Cruz Biotechnology Inc. SC-25310). The Sigma Fast Red kit includes levamosole to block endogenous alkaline phosphatase.

The specificity of all primary antibodies used was confirmed using controls incubated with nonimmune sera (equivalent protein concentration) of the species used to raise those antibodies. Positive and negative controls were performed for each antibody. Sections incubated with only secondary antisera enabled assessment of any nonspecific binding.

### Microscopy

Tissue sections were examined using an Olympus BX50 microscope with differential contrast optics and selected tissues were digitally imaged with a Spot RTse camera. Images were adjusted for contrast and labeled using Adobe Photoshop.

## RESULTS

### Functional Recovery after Olfactory Neurosphere-Derived Cell Transplantation

Transplantation of olfactory neurosphere-derived cells led to apparent restoration of normal resting heart rate after myocardial infarct (Fig. 3). These are highly inbred DA rats that have been “gentled” regularly. As well, they were sedated for the procedure under which circumstances they had a mean resting pulse of about 220. The transplanted group heart rate was (215.20 ± 13.26), that for the unoperated control (221.17 ± 5.71), and the infarct only group (347.75 ± 12.39). The difference between the transplanted and infarct alone groups was statistically significant ($p \leq 0.05$). There were trends for recovery of other measures in the transplanted group, but these did not achieve statistical significance of $p \leq 0.05$, with values of $p$ around 0.1. These measures were Ejection Fraction, Stroke Volume, Fractional Shortening, and E/A Ratio.

### Engraftment of Transplanted Cells

Sections from the left ventricle of transplanted animals and untransplanted controls were subjected to immunohistochemistry to detect GFP-labeled cells (Fig. 4). No GFP-labeled cells were detected in untransplanted animals. GFP-labeled cells were detected in all transplanted animals (n = 5). Some small, spherical cells were very intensely stained and located close to blood vessels. Larger cells were located in the gaps between myofibers. Another subgroup of cells was integrated within myofibers as single cells or in sometimes large clusters (Fig. 4A,C,G). Many of these GFP-labeled cells had the appearance of myocytes. Their identity was confirmed using antibodies to cardiac myofilaments (Fig. 4B,D,F,H). Darkly stained, GFP-labeled, donor-derived cells were subsequently double labeled for cardiac-specific phenotypic markers: striated muscle tropomyosin (Fig. 4B), cardiac troponin I (Fig. 4D), sarcomeric α-actin (Fig 4H), and GATA 4 (not shown). The sections shown are of ventricular myocardium and most cells (including donor-derived) appear positive for the muscle contractile apparatus proteins sarcomeric tropomyosin, cardiac troponin I, and sarcomeric α-actin. The Fast Red dye converted by the alkaline phosphatase–conjugated secondary antibody appears geometrically arrayed, consistent with the striated structure of myofibers of which these proteins are components in cardiac muscle. GATA 4 is a transcription factor expressed in developing cardiomyocytes and was only expressed sporadically throughout the section, sometimes associated with donor-derived cells (not shown).

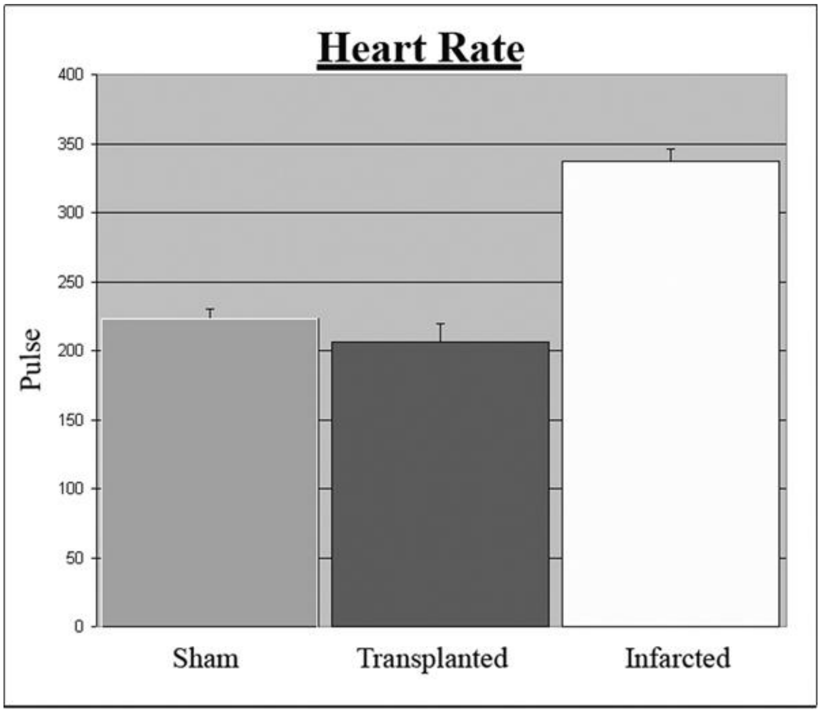

**FIGURE 3.** Comparison of basic cardiac physiology. Transplantation of olfactory neurosphere-derived cells led to restoration of normal resting heart rate after myocardial infarct. The infarct alone group differed significantly from the transplanted and sham groups using ANOVA ($p \leq 0.05$).

### DISCUSSION

In this study, we show that adult, olfactory mucosa–derived, neural progenitor cells can be induced to differentiate into cells resembling cardiomyocytes when transplanted into the infarcted rat heart. Transplantation of these cells led to apparent functional recovery indicated by restoration of normal resting heart rate. However, elevated heart rate is not commonly used as an indicator of heart damage. Rather, inability for a patient's heart to increase its rate in response to physical stress is an indicator of damage postinfarct[42,43,44,45]. However, it is thought that faster pulse is induced as a compensatory measure as animals attempt to maintain sufficient outflow[46,47]**.** It would seem, therefore, that these animals have not sustained too severe damage, and in a resting state are able to maintain marginally diminished functional performance by increasing heart rate. Donor-derived cells were detected 35 days after transplantation. Most of these were integrated into the myocardium and expressed cardiac muscle contractile proteins.

Neurosphere-derived cells from the mouse brain differentiated into the myocardium when transplanted into chick gastrulae[32]. This was confirmed in a similar experiment with neurosphere-derived cells from the human olfactory mucosa and noncultured cells dissociated from the mouse olfactory epithelium[3]. These experiments suggest that neural tissue–derived stem cells have the capacity to differentiate into mesodermal developmental fates, such as cardiac myocardium. The present data provide preliminary evidence for the efficacy of olfactory stem cells to repair the infarcted heart. They should be confirmed with larger numbers of animals and improved quantitation of echocardiographic dynamics, which has the potential to demonstrate the link between functional recovery and the presence of transplanted cells in the infarcted region.

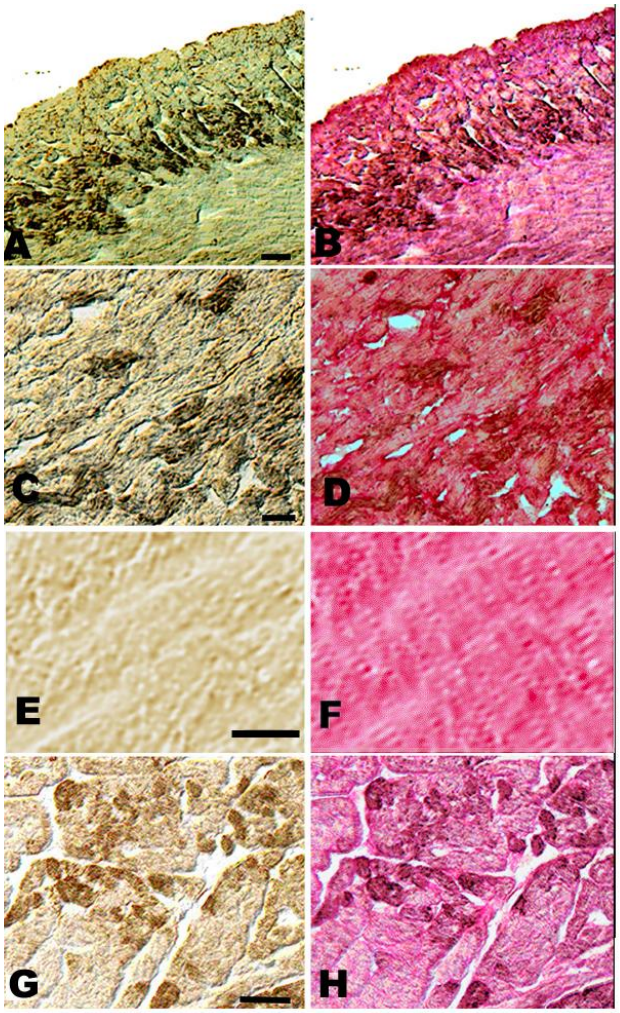

**FIGURE 4.** Immunohistochemistry identifying transplanted cells and cardiophenotypic proteins. Immunofluorescent techniques for antigen detection were not used due to the high degree of autofluorescence characteristic of fixed cardiac muscle that has led to some recent experimental misinterpretation. Immunohistochemistry was performed to identify transplant-derived cells expressing GFP (left panel: black/brown staining; A,C,G), and subsequently cardiophenotypic proteins (right panel, red staining). (B) Striated tropomyosin, (D) cardiac troponin I (H), sarcomeric α-actin. (E,F). Serial sections showing a negative (E, no primary antibody) and a positive control (F) for cardiac troponin I. Bar: 20 μm.

With the exception of heart rate, the functional data obtained are only at trend level significance. Animal numbers were necessarily limited by the Griffith Animal Ethics Committee until the procedures were established, initially involving high mortality. This limited statistically significant outcomes. It may be noted, in this respect, that the echocardiographers were volunteers and it was not possible to have the same operator for each animal tested. As well, the facility was unavailable due to unforeseen circumstances on two occasions, therefore limiting the number of animals tested. Nonetheless, the data suggest that transplanted animals retained better left ventricle function than infarcted, nontransplanted animals. This trend for reduced left ventricular function was apparent in several echocardiography parameters, such as Ejection fraction, Stroke Volume, Fractional Shortening, and E/A fraction. Fractional shortening assesses the ability of the ventricle to contract by relating the end-diastolic dimension to the end-systolic dimension of the left ventricle. A decrease in fractional shortening is associated with decreased left ventricular function in a number of myocardial diseases[48,49]. E/A ratio is the ratio of early/late diastolic peak flow velocity and is suggested to be a more sensitive indicator of myocardial impairment[11].

Importantly, histological analysis indicates that donor-derived cells were present in the infarcted heart 35 days after transplantation. They were integrated into the myocardium and many expressed myocardial contractile proteins. The number of donor cells surviving after transplantation could not be assessed. Although 500,000 cells were transplanted, many may not have had the capacity to differentiate into cardiac cells. Neurospheres are a mixed population of cells comprising unknown proportions of stem cells, neural progenitors, and differentiating neurons and glia[50]. When olfactory neurospheres are dissociated and propagated in different media, they express markers of neurons, glia, and oligodendrocytes in varying proportions[3]. In fetal calf serum, for instance, they consist of approximately 60% astrocytes, 20% neurons, and about 4% oligodendrocytes[3]. Within neurospheres, the majority of cells express nestin and may coexpress markers of these cells types[3]. The proportion of these with cardiomyogenic potential is unknown, but the present data indicate that this might be a productive area for further research. Rat hearts contain around $10^7$ myocytes[51], and a rough estimate of the infarct volume suggests that in the order of $2–5 \times 10^5$, cells would be susceptible to damage so the number of cells delivered was estimated to be appropriate.

Cardiomyocytes are specialized muscle cells that express subtly different isoforms of contractile proteins compared to skeletal muscle, although “specific” isoforms are seldom confined to one muscle type[52]. In the present study, we identified three contractile protein targets: α-tropomyosin (otherwise termed “striated”), which is only expressed in skeletal and cardiac muscle in the adult[53,54]; “sarcomeric” α-actins, with distinct isoforms for each type of muscle, but antisera have not distinguished cardiac and skeletal isoforms, so that the antibody used in our study recognizes both isoforms[55]; cardiac troponin I, described as the only cardiac-specific gene product and the antibody used recognizes only this cardiac-specific isoform[56,57,58,59]. As expected, we detected all three of these cardiac markers in myocytes of ventricular sections. All three were also observed in donor-derived, GFP-labeled cells, indicating that these cells differentiated along the cardiomyocyte lineage[60].

### CONCLUSION

Neural stem cells from the olfactory mucosa are multipotent, with the capacity to differentiate into developmental fates other than neurons and glia, with evidence of cardiomyocyte differentiation *in vitro* and after transplantation into the chick embryo. In this study, we show that adult, olfactory mucosa–derived, neural progenitor cells can be induced to differentiate into cells resembling cardiomyocytes when transplanted into the infarcted rat heart. Transplantation of these cells led to apparent functional recovery, indicated by restoration of heart rate. Donor-derived cells were detected 35 days after transplantation. Most of these were integrated into the myocardium and expressed cardiac muscle contractile proteins. These results suggest that olfactory mucosal stem cells could provide a basis for the development of autologous cell transplant therapy.

### ACKNOWLEDGMENTS

The authors wish to thank Bernadette Bellette and J. Kan for technical assistance.
